# Receptor Interactive Protein Kinase 3 Promotes Cisplatin-Triggered Necrosis in Apoptosis-Resistant Esophageal Squamous Cell Carcinoma Cells

**DOI:** 10.1371/journal.pone.0100127

**Published:** 2014-06-24

**Authors:** Yang Xu, Zhengwei Lin, Nan Zhao, Lanping Zhou, Fang Liu, Zbigniew Cichacz, Lin Zhang, Qimin Zhan, Xiaohang Zhao

**Affiliations:** 1 State Key Laboratory of Molecular Oncology, Cancer Institute & Hospital, Chinese Academy of Medical Sciences & Peking Union Medical College, Beijing, China; 2 Departments of Pharmacology and Chemical Biology, University of Pittsburgh Cancer Institute, Pittsburgh, Pennsylvania, United States of America; 3 Biodesign Institute, Arizona State University, Tempe, Arizona, United States of America; 4 Center of Basic Medical Sciences, Navy General Hospital, Beijing, China; Wayne State University School of Medicine, United States of America

## Abstract

Cisplatin-based chemotherapy is currently the standard treatment for locally advanced esophageal cancer. Cisplatin has been shown to induce both apoptosis and necrosis in cancer cells, but the mechanism by which programmed necrosis is induced remains unknown. In this study, we provide evidence that cisplatin induces necrotic cell death in apoptosis-resistant esophageal cancer cells. This cell death is dependent on RIPK3 and on necrosome formation via autocrine production of TNFα. More importantly, we demonstrate that RIPK3 is necessary for cisplatin-induced killing of esophageal cancer cells because inhibition of RIPK1 activity by necrostatin or knockdown of RIPK3 significantly attenuates necrosis and leads to cisplatin resistance. Moreover, microarray analysis confirmed an anti-apoptotic molecular expression pattern in esophageal cancer cells in response to cisplatin. Taken together, our data indicate that RIPK3 and autocrine production of TNFα contribute to cisplatin sensitivity by initiating necrosis when the apoptotic pathway is suppressed or absent in esophageal cancer cells. These data provide new insight into the molecular mechanisms underlying cisplatin-induced necrosis and suggest that RIPK3 is a potential marker for predicting cisplatin sensitivity in apoptosis-resistant and advanced esophageal cancer.

## Introduction

Esophageal cancer is the sixth most common cancer worldwide, and its highest incidence rates occur in Eastern Asia and Southern and Eastern Africa [1], [2]. The current standard of care for locally advanced esophageal cancer includes chemotherapy and radiotherapy without surgical treatment; chemotherapy consists of a combination of cis-diamminedichloroplatinum II (cisplatin) and 5-fluorouracil [3]. Apoptosis is well known to be the predominant form of cell death mediating chemotherapy and radiotherapy effectiveness [4], [5]. However, the upregulation of anti-apoptotic proteins and the downregulation of pro-apoptotic proteins often allow tumor cells to circumvent apoptosis, and become resistant to therapy during their evolution to malignancy [6]. Although cisplatin has been demonstrated to involve DNA binding, forming inter- and intra-stand covalent adducts, thus leading to apoptosis, accumulating evidence has shown that cisplatin-induced DNA adducts trigger both apoptosis and necrosis in cancer cells [7].

Apoptosis, as a process of programmed energy-driven, is characterized by caspase activity, nuclear condensation, degradation of cellular proteins and the formation of apoptotic bodies, with the maintenance of plasma membrane integrity. There are two core pathways to induce apoptosis, the extrinsic-death receptor pathway and the intrinsic-mitochondrial pathway. In contrast, necrosis is characterized by plasma membrane rupture, swollen organelles and release of cellular proteins into the extracellular microenvironment. With the discovery of key mediators of necrotic cell death such as RIPK1 and RIPK3, accumulating data show that necrosis is also programmed cell death. Recent evidence shows that caspase-8- and FADD-deficient mice die at embryonic stage 10.5; which is rescued by co-deletion of RIPK1 or RIPK3. This indicates that inhibition of the caspase-8-dependent apoptotic pathway triggers RIPK3-dependent necrosis, leading to death during embryonic development [8], [9]. Because tumor cells evolve various strategies to evade apoptosis during tumorigenesis, necrosis can be found in tumor tissues during chemotherapy and radiotherapy [10], [11]. Increasing evidence indicates that the process of cancer transformation is accompanied by a shift from apoptosis to necrosis. Cancer cells can die by different cell death modes including necrosis in response to genotoxic drugs [12]. It has also been found that treatment of tumor with cisplatin showed significantly released levels of HMGB and caused necrosis, particularly in skin tumors [13]. The role of necrotic cell death in chemotherapy has been increasingly appreciated [14], [15]. Nevertheless, the mechanisms of programmed necrosis induced by cisplatin remain largely unknown.

Recent evidence has demonstrated that TNFα triggers programmed necrosis following experimental inhibition of caspase activation in a number of cell types [16]. RIPK3 has been identified in a genome-wide siRNA screen as a critical necrosis mediator which switches the cell fate from TNFα-induced apoptosis to necrosis [17], [18]. The execution of programmed necrosis requires the functions of RIPK3 and RIPK1, and can be blocked by the RIPK1 kinase inhibitor necrostatin and the RIPK3 inhibitor necrosulfonamide (NSA), especially when the apoptotic pathways are suppressed [17], [19], [20], [21]. RIPK1 and RIPK3 are both important components of the necrosome, a death-signaling complex that is required for necrosis in response to TNFα. Mixed lineage kinase domain-like protein (MLKL) has recently been identified as another essential member of the necrosome complex, and a key downstream mediator of the RIPK3. This large multi-protein complex has been characterized in the presence of caspase inhibitors and a SMAC mimetic [22], [23]. Phosphorylation of both RIPK1 and RIPK3 stabilizes their association within the death-signaling complex and results in the production of reactive oxygen species (ROS) in certain cells by activating enzymes in metabolic pathways [24], [25]. The expression of RIPK3 has been detected in a variety of cancer cell lines, which correlates with responsiveness to necrosis induction [17]. However, whether RIPK3 is associated with the cisplatin-induced necrosis is largely unknown.

In this study, we demonstrated that cisplatin selectively induces necrosis in KYSE140, an esophageal squamous carcinoma (ESCC) cell line deficient in SMAC, a pro-apoptotic protein. Cisplatin triggers necrosome formation through autocrine production of TNFα, leading to necrosis. Furthermore, inhibition of RIPK activity by Nec-1 or knockdown of RIPK3 protein with siRNA largely rescued cisplatin-induced necrosis. Moreover, microarray analysis identified an anti-apoptotic molecular expression pattern in esophageal cancer cells in response to cisplatin. Our data thus provide new insight into the molecular mechanisms underlying cisplatin-induced necrosis in esophageal cancer chemotherapy.

## Materials and Methods

### Cell culture and drug treatment

The esophageal cancer cell lines KYSE140 and KYSE410 were obtained from Dr. Yutaka Shimada at Hyogo College of Medicine[26]. EC0156 was established in our lab (19). All cell lines were grown in RPMI supplemented with 10% FBS, 100 U/ml penicillin, and 100 µg/ml streptomycin at 37°C in 5% CO_2_. Cisplatin was purchased from the institutional pharmacy (QILU Pharmaceutical Co., China). Cells were grown in 96-well plates to 20–30% confluence before treatment with anticancer drugs. After cisplatin treatment for 24 or 48 h, cell death was analyzed using a MTT assay.

### Transfection and small interfering RNA (sh RNA) knockdown

For RIPK3 and SMAC knockdown, KYSE140 cells grown in a 6-well plate were transfected with the pPuro-shRNA-RIPK3 and pSilence-shRNA-Smac plasmids using Lipofectamine (Invitrogen, USA) according to the manufacturer's instruction [17] [27]. For RIPK3 overexpression, KYSE410 cells were transfected with PCI-RIPK3 vector using Lipofectamine (Invitrogen, USA) [17]. The nonspecific small interfering RNA (siCtrl) oligonucleotides and siRNA oligonucleotides targeting RIPK1 were obtained from Life Technologies Co. (Ambion, USA). All siRNAs were transfected into KYSE140 cells using the Lipofectamine Plus reagent (Invitrogen) according to the manufacturer's protocol.

The following abbreviations are used throughout the paper: RIPK3-KD, a selected KYSE140 clone with reduced RIPK3 expression (14); PCI-RIPK3, a selected KYSE410 clone with exogenous overexpression of RIPK3 from the PCI vector; KYSE140-vec, KYSE140 cells transfected with the empty vector.

### Measurement of TNFα and LDH in culture media by ELISA

Cells grown in 10 cm dishes were treated at the indicated time as described in figure legends. The culture media were collected, and the concentrations of TNFα and LDH were determined with a TNFα ELISA Kit (Invitrogen) and a lactate dehydrogenase (LDH) ELISA Kit (Abcam) according to the manufacturers' protocols.

### Detection of cell death and viability

Cell death was detected by (i) nuclear morphological changes examined under a microscope and (ii) the annexin V and propidium iodide (PI) staining coupled with flow cytometry, as described previously (19). Cell viability was assessed by the MTT (AMRESCO, USA) assay using a Model 680 microplate reader (Bio-Rad, CA) to quantify absorbency value according to the manufacturer's instruction. For the colony formation assay, cells were seeded in 6-well plates at a density of 5×10^5^ cells per well, treated with cisplatin for 12 h, and then cultured with fresh, drug-free RPMI supplemented with 10% FBS. After 14 days, cells were stained with 0.25% crystal violet (AMRESCO) for 20 min. All experiments were repeated at least three times.

### Immunoprecipitation and western blot analysis

Immunoprecipitation of RIPK1 and MLKL was performed according to a previously described protocol [28]. Briefly, cells cultured in 10-cm dishes were lysed for 30 min on ice in lysis buffer containing 1% (w/v) Triton X-100, 0.15 M NaCl, 30 mM Tris-HCl (pH 7.5) and protease inhibitors (Roche, Germany). The lysates were sonicated and centrifuged at 10,000 *g* for 15 min at 4°C. One milligram of extracted protein in lysis buffer was incubated overnight with 10 µg rabbit anti-RIPK1 or anti-MLKL antibody followed by incubation with 20 µl Dynabeads Protein G (Invitrogen, USA) for 2 h at 4°C and three washes with lysis buffer. The beads were directly boiled in 1% SDS loading buffer for 5 min. Subcellular fractions, including nuclear, membrane and cytosolic fractions, were prepared using the proteoExtract sub-cellular proteome extraction kit (Calbiochem, USA) according to the manufacturer's instructions.

Western blot analysis was performed as described previously [27], using the following antibodies: anti-caspase-9, anti-caspase-3, anti-Fos, anti-RIPK1, and anti-survivin antibodies from Cell Signaling Technology (Beverly, MA); anti-RIPK3 and anti-SMAC antibodies from Abcam (Cambridge, MA); anti-MLKL, anti-cytochrome C and anti-Bcl-2 antibodies from Santa Cruz Biotechnology.

### Microarray analysis

Total RNA was isolated from untreated and cisplatin-treated KYSE140 cells using an RNeasy mini kit (Qiagen). The quality of the total RNA was determined using a Nanodrop spectrophotometer (ND-2000; Thermo Fisher Scientific). RNA with an A260/A280 absorbance ratio ranging from 1.8 to 2.0 was used for cDNA synthesis. Gene expression profiles were analyzed on a GeneChip Human Genome U133 Plus 2.0 array (Affymetrix, Santa Clara, CA), which contains 54,000 probe sets representing approximately 47,000 genes. Microarray hybridization was performed at 45°C with rotation for 16 h using an Affymetrix GeneChip Hybridization Oven 640. The stained GeneChip probe array was scanned with the GeneChip Scanner 3000 7G (Affymetrix) at 570 nm. The signal intensity of the gene expression was analyzed to generate CEL files using the default setting of Affymetrix GeneChip Command Console 3.2 (AGCC) Software. The Affymetrix Microarray Suite 5.0 (MAS5) and the Robust Multi-array Average (RMA) algorithm were used for the expression summary and signal calculation of the GeneChip Human Genome U133 2.0 data, respectively [29]. In a comparison analysis, the two-classes unpaired method from the Significance Analysis of Microarray software (SAM, version 3.02) was used to compare the significantly differentially expressed genes between the untreated and cisplatin-treated KYSE140 cells. Differentially expressed genes were selected based on a >2.0-fold change and a q value <5%. The Entrez gene identifiers from the genes were used to perform enrichment analysis using the Database for Annotation, Visualization and Integrated Discovery (DAVID) [30]. Geo series number is GSE56769.

### Real-time PCR Analysis

Total RNA was isolated from the untreated and cisplatin-treated KYSE140 cells using an RNeasy mini kit (Qiagen, USA). The first-strand complementary synthesis reaction was performed using the SuperScript III Reverse Transcriptase kit (Invitrogen, USA). Primers were designed with Primer-Blast software (http://www.ncbi.nlm.nih.gov/tools/primer-blast/). Amplification reactions were conducted using the SsoFast EvaGreen Supermix with a CFX 96 real time system (Chemoscience, USA). The complete list of gene-specific real-time primers in this study is available in Table S3. GAPDH served as an internal control to normalize the loading of the template cDNA. Each experiment was repeated at least twice, and the fold change in gene expression was assessed using theΔCt method [31].

### Flow cytometric analysis

KYSE140 cells were treated with 10 µM cisplatin for 6 and 12 h, then incubated with 2′,7′-dichlorofluorescin diacetate (H2DCF-DA) for 20 min after washing with PBS. ROS activation was determined and analyzed using flow cytometry (EPICS ELITE ESP, USA) to calculate the percentage of ROS-positive cells according to the manufacturer's protocol.

### Transmission electron microscopy

KYSE140 cells cultured in chamber slides were treated with 10 µM cisplatin for 24 h, then fixed for 2 h with 2.5% glutaraldehyde in 0.1 M sodium cacodylate buffer (pH 7.4), further fixed in osmium tetroxide solution for 1 h, and then subjected to electron microscopy analysis with a Hitachi 7650 electron microscope.

### Ethics statement

All experimental procedures for the use of animals were previously reviewed and approved by the institutional animal care and use committee (IACUC) at the Cancer Hospital of Chinese Academy of Medical Science (Permit Number: NCC2013A012). All surgery was performed under sodium pentobarbital anesthesia, and all efforts were made to minimize suffering.

### *In vivo* xenograft assay

Animal experiments were carried out as previously described [27]. Briefly, 5×10^6^ parental or PCI-RIPK3-D8 cells were suspended in 100 µl PBS and injected subcutaneously into the right flank of female nude mice (*n* = 5). Tumor size was measured every 2–3 days with digital calipers. Tumor volume was calculated using the formula: 0.5×*a*×*b* ^2^, where *a* is the length of the tumor, and *b* is the width.

### Statistical analysis

Statistical analysis was performed using GraphPad Prism 5 software. Statistical comparisons were made using SPSS version 17.0. *P*-values <0.05 were considered statistically significant.

## Results

### Cisplatin induces non-apoptotic cell death in SMAC-deficient ESCC cells

**Figure 1:**
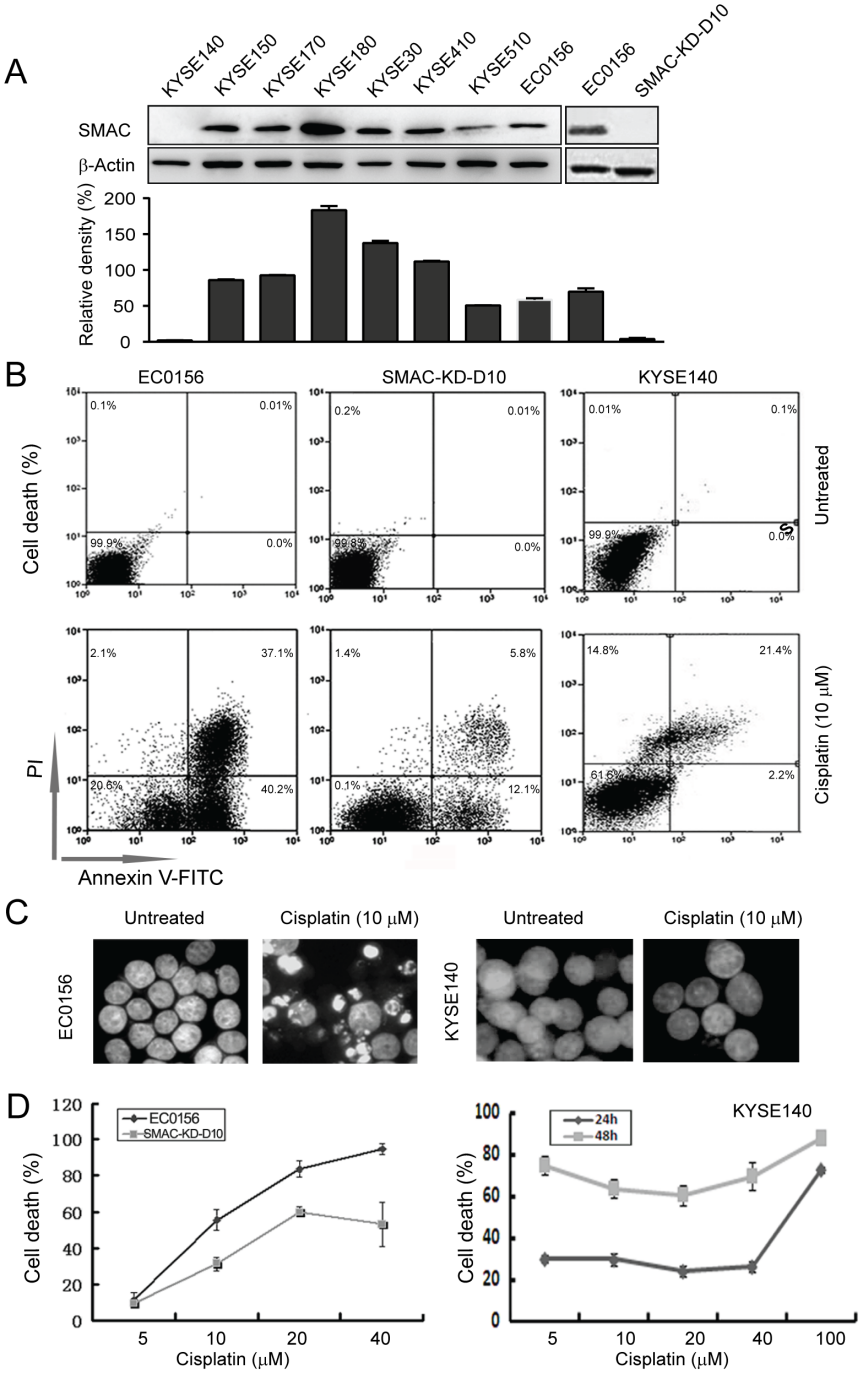
Cisplatin-induced cell death in ESCC cells. (A) Total cellular protein extracts from each of the nine esophageal cancer cell lines were subjected to western blot analysis for SMAC; SMAC-KD-D10 shows stable knockdown of SMAC. The levels of SMAC protein were quantified, normalized to β-Actin, and are shown in the lower bar graph. (B) Apoptosis was observed in the KYSE140, EC0156, and SMAC-KD-D10 cells after treatment with cisplatin for 24 h, using annexin V/propidium iodide staining and flow cytometry. (C) Nuclei were observed following nuclear staining with DAPI. More apoptotic nuclei appeared in the EC0156 cells after exposure to cisplatin for 24 h, but few apoptotic nuclei were observed in the treated KYSE140 cells. (D) Dose-dependence curve for the KYSE140, EC0156, and SMAC-KD-D10 cells after cisplatin treatment for 24 h and 48 h.

**Figure 2:**
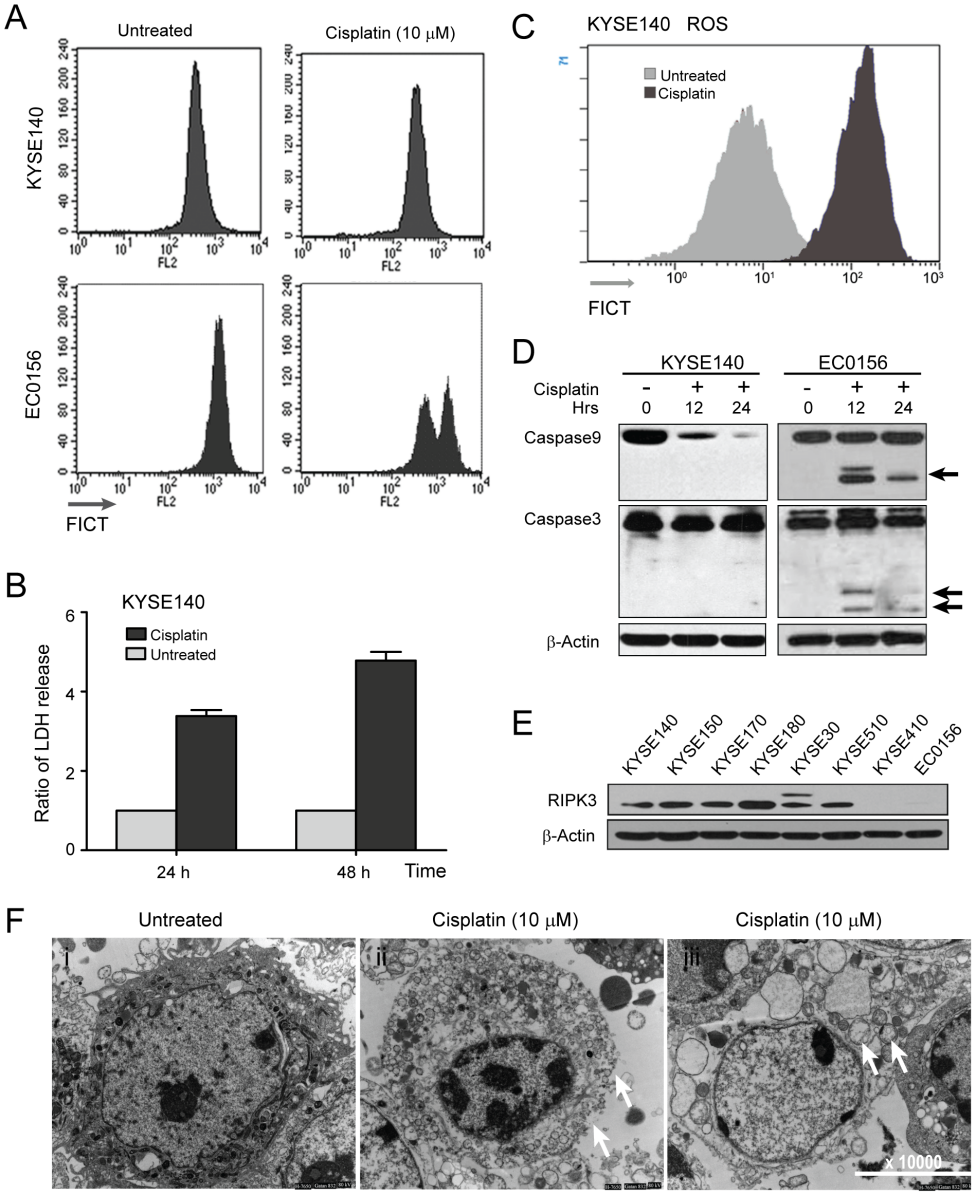
Cisplatin triggers programmed necrosis in KYSE140 cells. (A) Mitochondrial membrane potentials of KYSE140 and EC0156 cells were determined using Mito Tracker Red CMXRos staining and analyzed by flow cytometry after cisplatin treatment for 24 h. (B) Cellular necrosis was measured as LDH release after cisplatin treatment for 24 h and 48 h. (C) KYSE140 cells were treated as indicated for 12 h. The cells were stained with H2DCF-DA for 20 min, and then ROS levels were measured using flow cytometry. All experiments were performed at least three times. (D) KYSE140 and EC0156 were treated with 10 µM cisplatin for 12 or 24 h. Caspase activation was assessed by western blotting. Arrowheads indicate caspase cleavage fragments. (E) Total cellular protein extracts from each of the eight esophageal cancer cell lines were subjected to western blot analysis for RIPK3. β-Actin as a loading control. (F) Transmission electron microscopy of the KYSE140 cells after cisplatin treatment. Membrane integrity was noted in the control cells, and the collapse of the membrane and the swelling of the cellular organelles were noted in the cells treated with cisplatin.

Our previous work revealed that SMAC modulates chemosensitivity and is essential for drug-induced apoptosis in esophageal cancer [27]. In this study, we first asked whether the innate SMAC deficiency facilitates cisplatin resistance in ESCC cells. As shown in Figure 1A, western blot analysis of SMAC in ESCC cell lines showed that 7 of the 8 lines express SMAC, except for KYSE140. We chose KYSE140 as an innate SMAC-deficient cell model to investigate cisplatin resistance. The sensitivities of EC0156 with SMAC knockdown (SMAC-KD) and KYSE140 cells to cisplatin were examined using annexin V-FITC and propidium iodide. Intriguingly, we found that KYSE140 cells that are endogenously SMAC-deficient were still sensitive to cisplatin-induced cell death in comparison to the SMAC-KD cells (Figure 1B and 1D). To investigate whether KYSE140 cells die via apoptosis, cells were examined for apoptotic morphology. Substantial nuclear fragmentation and mitochondrial membrane potential were detected in the positive control EC0156 cells, but not in the KYSE140 cells after cisplatin treatment (Figure 1C and 2A), suggesting that cisplatin induces non-apoptotic cell death in the KYSE140 cells.

These observations prompted us to further investigate the type of cell death induced by cisplatin in KYSE140 cells using several approaches. First, we analyzed the release of lactate dehydogenase (LDH), a characteristic feature of necrotic cell death [32]. We noticed that the release of LDH was apparently increased at 24 and 48 h after cisplatin treatment (Figure 2B). Next, we tested the ROS production of the cisplatin-treated KYSE140 cells. ROS production increased in response to cisplatin in KYSE140 cells (Figure 2C). Furthermore, monitoring activation of the caspase cascade by western blot analysis revealed little cleavage of caspase-9 and caspase-3 after cisplatin treatment in KYSE140 cells, whereas caspase cleavage was observed in the control EC0156 cells, further indicating a non-apoptotic form of cell death in KYSE140 cells (Figure 2D). Further analysis using transmission electron microscopy (TEM) showed a substantial percentage of cisplatin-treated KYSE140 cells had discontinuous cytoplasm membranes and swollen mitochondria, but lacked typical apoptotic features such as plasma membrane blebbing (Figure 2F-i and 2F-iii). Collectively, these data indicates that cisplatin predominately induces non-apoptotic and caspase-independent cell death in KYSE140 cells.

### The molecular mechanisms of cisplatin-induced necrosis in KYSE140 cells

**Figure 3:**
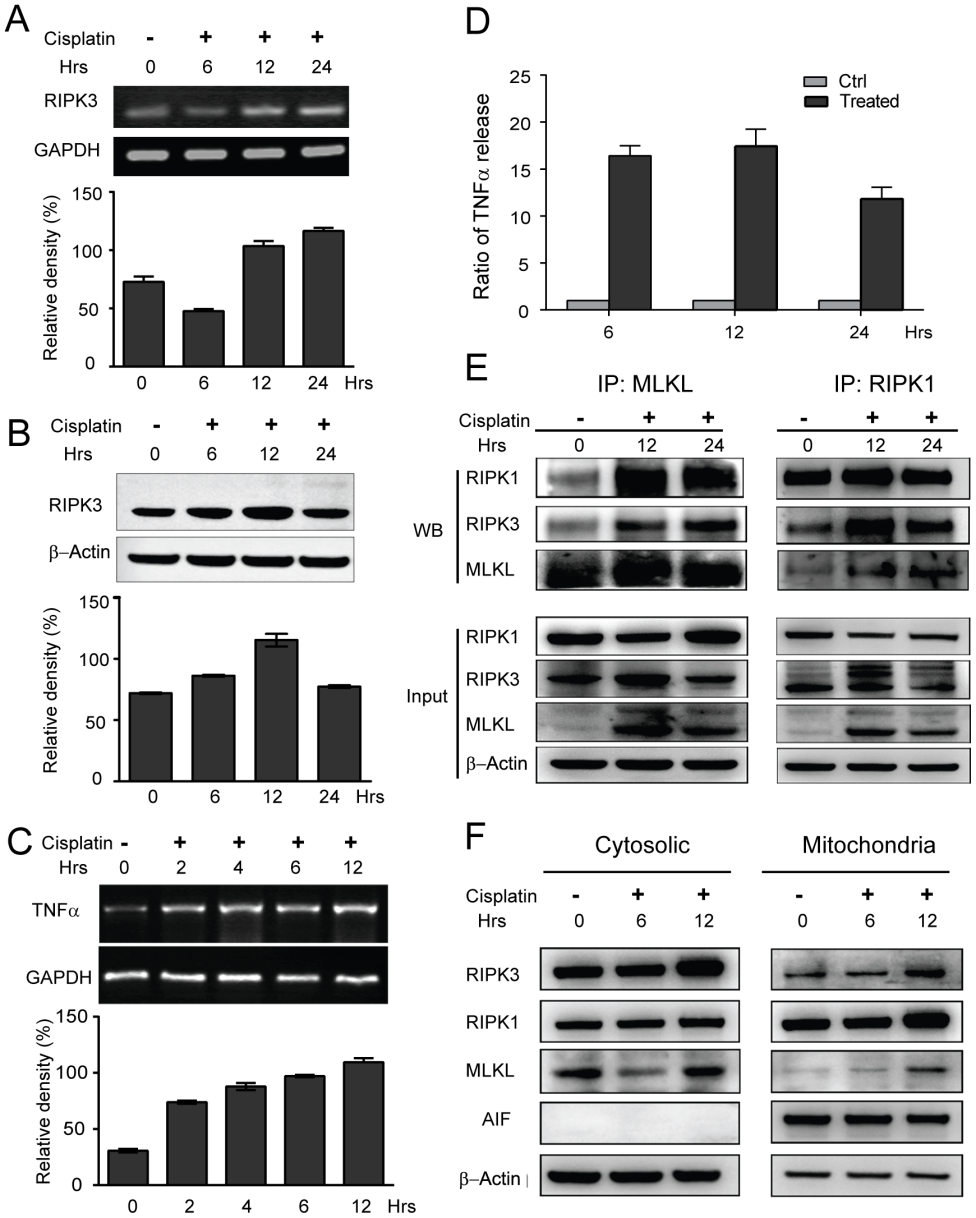
Cisplatin triggers the assembly of necrosome via autocrine production of TNFα in KYSE140 cells. (A) KYSE140 cells were treated with 10 µM cisplatin for 6, 12, or 24 h, and the RIPK3 mRNA level was determined by RT-PCR. The relative density was compared to the GAPDH control. (B) The RIPK3 protein expression was determined by western blot analysis. The levels of RIPK3 protein were quantified and normalized to β-Actin. (C) Cisplatin promotes TNFα transcription. KYSE140 cells were treated with 10 µM cisplatin for the indicated time points, and the TNFα mRNA levels were analyzed by RT-PCR. The relative density was compared to the GAPDH control. (D) Cisplatin promotes autocrine production of TNFα. KYSE140 cells were treated with cisplatin for the indicated time points, and the level of TNFα secretion in the supernatant was measured by ELISA. (E) RIPK3 interacts with RIPK1 and MLKL. After treatment with cisplatin for 12 and 24 h, RIPK1 and MLKL were immunoprecipitated using anti-RIPK1 or anti-MLKL antibodies. RIPK1, RIPK3 and MLKL were detected by western blot analysis. (F) KYSE140 cells were treated with 10 µM cisplatin for 6 or 12 h. Mitochondrial and cytosolic fractions were isolated from the treated cells and analyzed for the indicated proteins with western blotting. AIF was used as a control for mitochondrial fraction and loading.

RIPK3 has recently been identified as a critical modulator in TNFα–induced necroptosis [17], [33]. We examined the expression levels of RIPK3 in several ESCC cell lines. As shown in Figure 2E, western blot analysis showed that 6 of the 8 lines, including KYSE140, expressed RIPK3, except for EC0156 and KYSE410 cells. We found that the transcription and protein expression of RIPK3 gradually increased from 6–24 h after cisplatin treatment compared to the untreated cells (Figure 3A&B). Autocrine production of TNFα has recently been recognized as a critical signal for the induction of necroptosis in response to zVAD and Heme [34], [35]. We tested whether autocrine production of TNFα is involved in cisplatin-induced necrotic cell death in KYSE140 cells by analyzing TNFα mRNA levels using RT-PCR at the indicated time points after cisplatin treatment. A higher level of TNFα mRNA level was detected in cisplatin-treated KYSE140 cells than in control cells (Figure 3C). ELISA analysis detected a low basal level of TNFα, approximately 3 pg/ml, in the media of KYSE140 cells. The TNFα level was profoundly increased by over 15-fold upon cisplatin treatment for 6 or 12 h, suggesting that cisplatin treatment enhances the transcription of TNFα and autocrine production of TNFα in the media (Figure 3D). These results are consistent with recent studies in which autocrine production of TNFα was required for zVAD-induced cell death in L929 cells [18], [34].

It is known that functional necrosomes, formed by the binding of RIPK3 to the RIPK1 kinase and the mixed lineage kinase domain-like protein (MLKL), play key roles in TNFα-induced necrosis [36], [37]. Therefore, we sought to determine whether cisplatin triggered the assembly of necrosome served as a cytosolic signaling platform to initiate necrosis in KYSE140 cells. Anti-RIPK1 and anti-MLKL antibodies were used to immunoprecipitate the endogenous RIPK3. The RIPK3/RIPK1/MLKL complex was only weakly present in the untreated cells, but the level of necrosomes markedly increased after cisplatin treatment (Figure 3E). To verify the subcellular localization of the necrosome complex, we also analyzed sub-cellular fractions of the cisplatin-treated cells, including the cytosolic and the membrane/mitochondria protein fraction (Figure 3F). RIPK3 was enriched in the cytosolic fraction and the membrane/mitochondria fraction after 12 h of cisplatin treatment. RIPK1 and MLKL were also slightly enriched in the membrane fraction after cisplatin treatment. Taken together, these data indicate that cisplatin triggers the recruitment of the necrosome to mitochondrial membranes, which involves autocrine production of TNFα.

### RIPK3 and RIPK1 are required for cisplatin-induced necrosis

**Figure 4:**
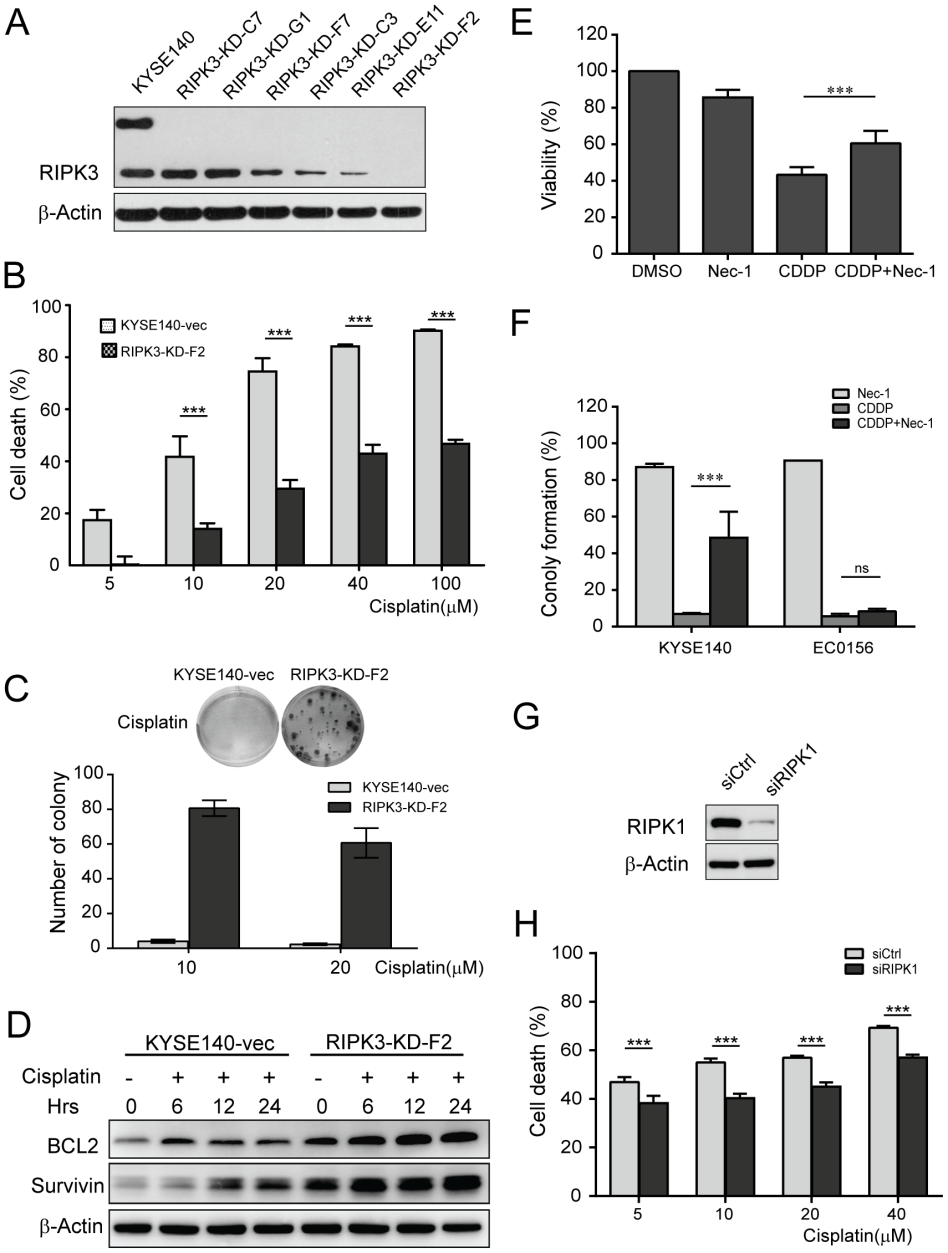
RIPK3 is required for cisplatin-induced necrosis. (A) Effects of RIPK3 knockdown were determined using western blot analysis, β-Actin as a loading control. (B) RIPK3-KD-F2 clones and the parental cells were treated with 10 µM cisplatin for 24 h. Cell viability was assessed by MTT assay. The data represent the mean percentage ± SD of cell death compared to parental cells. (C) The long-term viability of the cells was determined after cisplatin treatment for 6 h in RIPK3-KD cells using the colony formation assay. (D) Western blot analysis of Bcl-2 and survivin in the parental and RIPK3-KD-F2 cells after treatment with 10 µM cisplatin for 6, 12, or 24 h. β-Actin was used as a loading control. (E) KYSE140 cells were pre-incubated for 1 h with 20 µM Nec-1 or DMSO and then treated with 10 µM cisplatin for 24 h. Cell viability was determined using an MTT assay and is expressed as percentage of DMSO control. (F) KYSE140 and EC0156 cells were pre-incubated for 1 h with 20 µM Nec-1 or DMSO and then treated with 10 µM cisplatin for 24 h before medium was replaced with fresh medium. Colonies were identified using crystal violet staining and counted under the microscope. (G) Effects of RIPK1 knockdown were analyzed by western blotting, β-Actin as a loading control. (H) KYSE140 cells with RIPK1 siRNA were treated with the indicated concentrations of cisplatin for 24 h. The data represent the mean percentage ± SEM of cell death versus control cells.

Based on the finding that cisplatin triggers the formation of necrosome, we asked whether cisplatin-induced cell death depends on RIPK1 and RIPK3. RIPK3 was knocked down by shRNA vectors in KYSE140 cells. Efficiency of RIPK3 knockdown was confirmed by western blot analysis (Figure 4A). Knockdown of RIPK3 (RIPK3-KD) markedly suppressed cisplatin-induced cell death and considerably increased colony formation upon cisplatin treatment compared with control vector cells (Figure 4B and C). We also found that Bcl-2 and survivin were significantly upregulated in the RIPK3-KD KYSE140 cells in response to cisplatin compared to the parental cells (Figure 4D). Our data show that RIPK3 is required for cisplatin-induced cell death in KYSE140 cells.

It has been reported that necrostatin-1 (Nec-1), a RIPK1-specific inhibitor, can block necroptosis [38]. We tested the effects of Nec-1 on cisplatin-induced death in KYSE140 cells. Importantly, Nec-1 significantly inhibited the cisplatin-induced loss of cell viability (Figure 4E). Notably, Nec-1 also significantly protected clonogenic survival of KYSE140 cells after cisplatin treatment (Figure 4F). We also used siRNA to knock down RIPK1 to test the requirement for necrosis in cisplatin-induced cell death. Efficiency of siRNA-mediated knockdown of RIPK1 protein was confirmed by western blot analysis (Figure 4G). RIPK1 knockdown profoundly rescued cisplatin-induced cell death (Figure 4H), indicating that RIPK1 is required for cisplatin-induced cell death. These results indicate that both RIPK1 and RIPK3 are required for cisplatin-triggered necrosis and for protection against long-term clonogenic survival.

### Gene expression changes in the cisplatin-treated KYSE140 cells

**Figure 5:**
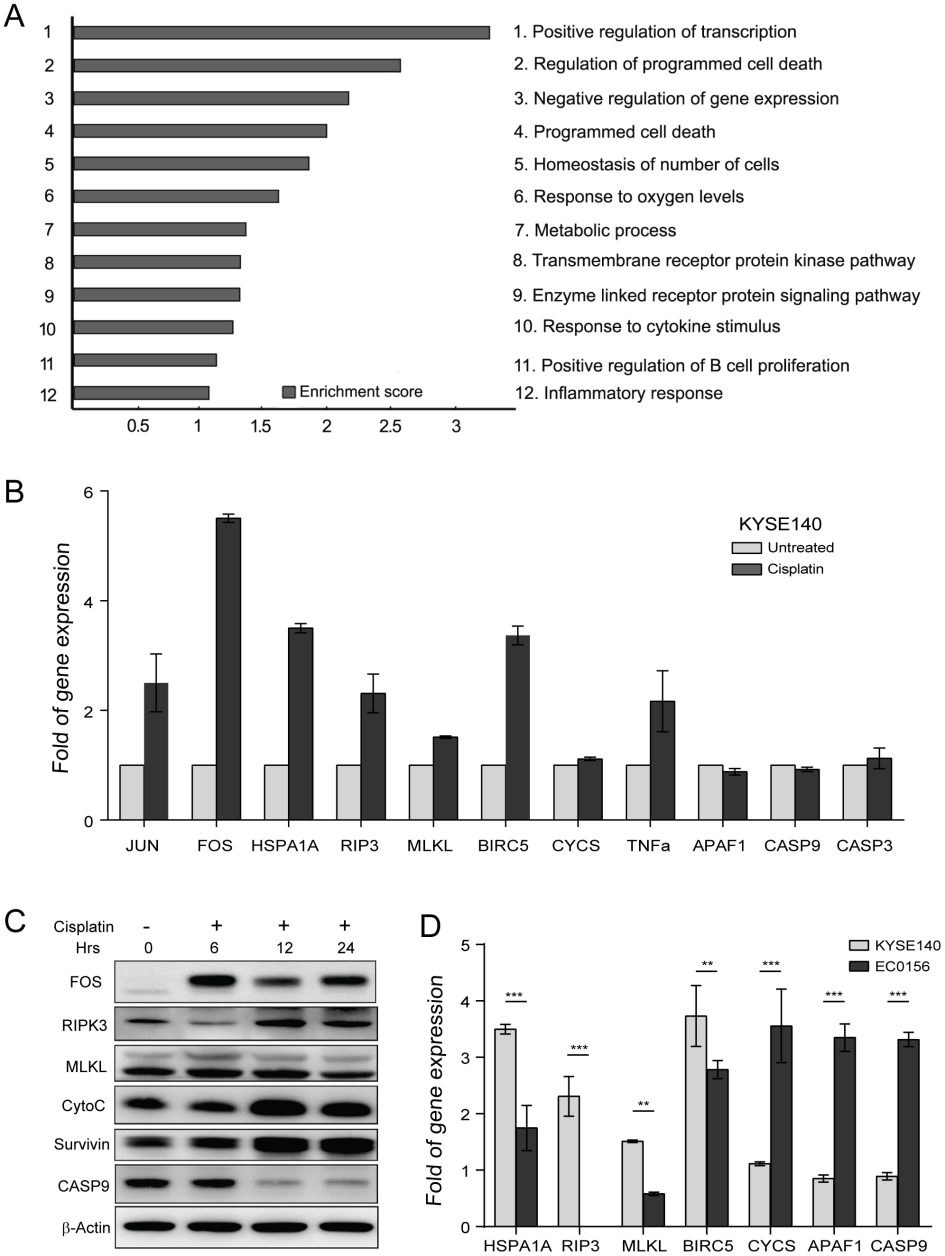
Clustering display of the microarray data after cisplatin treatment. (A) RNA was extracted using an RNA isolation kit for microarray analysis after cisplatin treatment for 6 h. The up-regulated genes were functionally classified based on their biological process using the DAVID functional annotation clustering tool. (B) The mRNA levels of cell-death-associated genes in KYSE140 cells treated with 10 µM cisplatin for 6 h were determined by real-time RT-PCR. The data represent the mean ± SEM of relative mRNA levels versus untreated cells. (C) Protein levels of FOS, RIPK3, MLKL, Cytochrome c, survivin and caspase-9 in KYSE140 cells after treatment with 10 µM cisplatin for the indicated time points were analyzed by western blotting. (D) The mRNA levels of cell-death-associated genes in KYSE140 and EC0156 cells treated with 10 µM cisplatin for 6 h were determined by real-time PCR. **, *P*<0.05; ***, *P*<0.01.

To further investigate the mechanism of cisplatin-induced necrosis in the KYSE140 cells, the Affymetrix Human Genome U133 plus 2.0 arrays were used to analyze mRNA changes in cisplatin-treated KYSE140 cells. The array data were analyzed using the Significance Analysis of Microarrays (SAM 3.02) software. The cutoff criteria for significantly differentially expressed genes were set as a ratio of >2-fold difference in expression and an adjusted *P* value of <0.05. In total, 531 genes fulfilled the stringent cutoff criteria; 94 genes were upregulated, whereas 437 genes were downregulated in the cispltin-treated group. Within the upregulated genes, we grouped similar annotation terms into clusters using DAVID functional annotation clustering to measure the relationships between the annotation terms and their co-association with the genes [30]. Notably, the upregulated genes were predominantly involved in the regulation of transcription, programmed cell death, response to changes in oxygen levels, and response to cytokine stimulus (Figure 5A). The complete list of upregulated and downregulated genes referenced to the canonical biological pathways is available in Table S1 & S2. To better understand how the KYSE140 cells are resistant to apoptosis induction after cisplatin treatment, genes associated with programmed cell death, including *FOS, JUN, HSPA1A, TNFα, RIPK3, MLKL, BIRC5, CYCS, APAF1, CASP9* and *CASP3*, were selected for validation of the microarray results by real-time PCR and western blot analyses. The mRNA levels of *FOS*, *JUN*, *HSPA1A* and *BIRC5*, which are associated with apoptotic resistance, were significantly elevated in the KYSE140 cells after treatment with cisplatin for 6 h. Furthermore, the mRNA levels of *TNFα, RIPK3* and *MLKL*, which are associated with necrosis, were greatly increased in the cisplatin-treated KYSE140 cells (Figure 5B). Additionally, the mRNA levels of *APAF1*, *CASP9* and *CASP3*, which are associated with apoptosis, were either unchanged or partially decreased following cisplatin treatment. Therefore, transcript analysis showed that cisplatin treatment facilitates transcription of necrosis-associated genes but not apoptotic genes in KYSE140 cells.

The expression levels of FOS, CASP9, RIPK3, MLKL, CYCS and survivin were further evaluated using western blot analysis (Figure 5C). The FOS protein level was markedly increased in the KYSE140 cells after cisplatin treatments for 6, 12 and 24 h. The RIPK3, CYCS and survivin expression levels were also higher in the cisplatin-treated cells. Furthermore, we examined whether cisplatin-induced cell death depended on differential gene expression in KYSE140 and EC0156 cells. Induction of RIPK3 and MLKL was observed in KYSE140 cells and reduced in EC0156 cells, which undergo apoptosis after cisplatin treatment [19]. In contrast, massive induction of CYCS, APAF1 and CASP9 occured in EC0156 but not in KYSE140 cells (Figure 5D). Together, these data demonstrate that cisplatin triggers necrosis in KYSE140 cells by increasing the expression of necrosis-associated genes as well as anti-apoptotic genes.

### Cisplatin-induced necrosis is enhanced by RIPK3 overexpression in cells with apoptosis blocking

**Figure 6:**
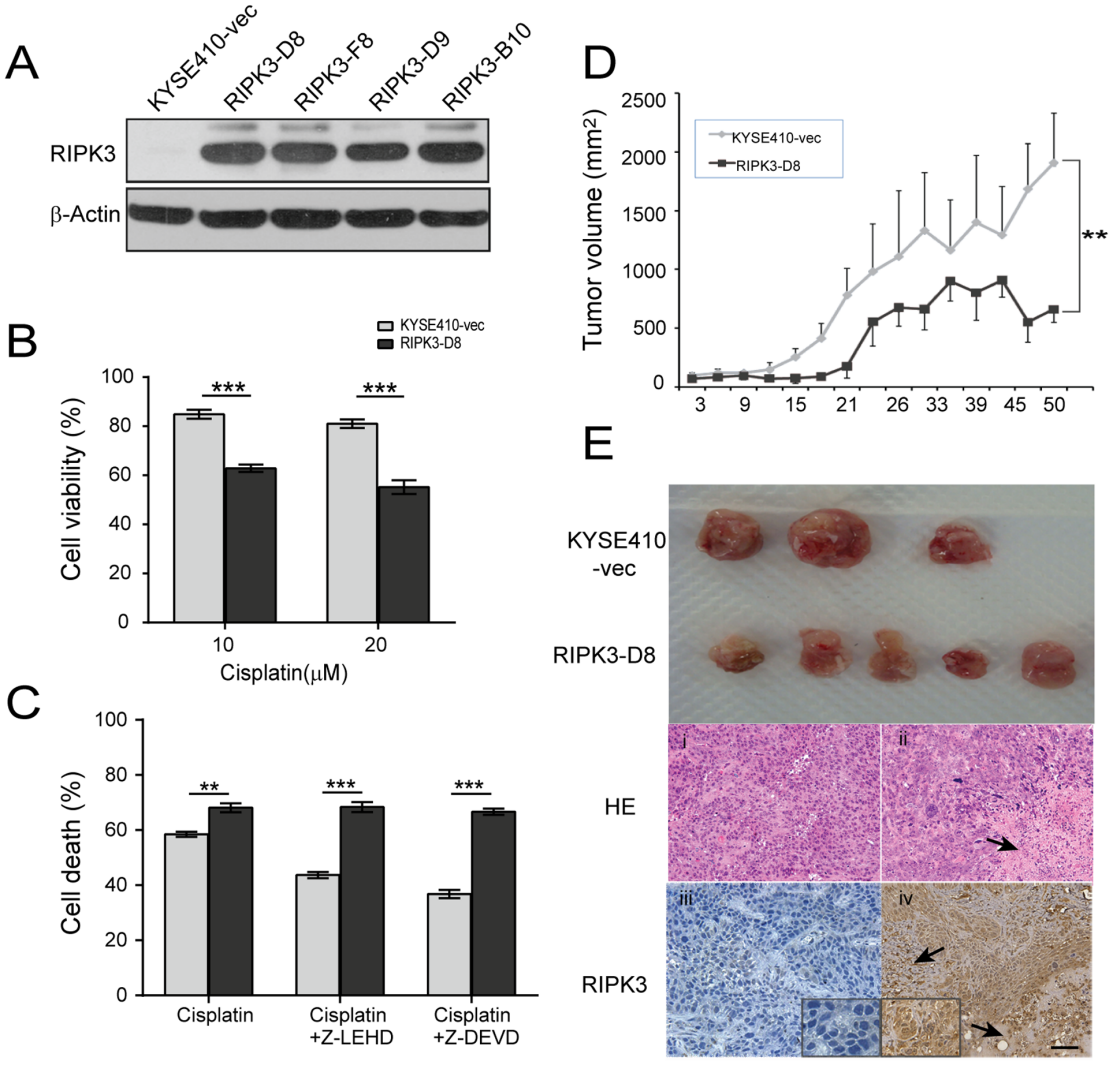
RIPK3 overexpression inhibits tumor growth. (A) RIPK3 overexpression was analyzed with western blot analysis in the stable KYSE410 clones. (B) RIPK3 overexpression clones and the parental cells were treated with 10 µM or 20 µM cisplatin for 24 h. Cell viability was assessed by flow cytometry. The data represent the mean percentage ± SD of viable cells compared to parental cells. (C) KYSE410-vec and RIPK3-D8 clone cells were treated with 10 µM cisplatin combined with a caspase inhibitor, z-LEHD (5 µM) or z-DEVD (5 µM), for 24 h. Cell viability was determined using an MTT assay. (D) Tumor volume was measured every 2 to 3 days after treatment. RIPK3-D8 clone cells decreased the growth of the xenograft tumors compared to the control group (*P*<0.001). (E) Reduced tumor growth in the RIPK3-overexpressing cells compared to the parental KYSE410 cells. The parental and RIPK3-D8 cells were suspended in 0.1 ml of PBS and injected into nude mice to establish xenograft tumors. The bottom panels show H&E staining and the RIPK3 expression determined by immunostaining for xenograft tissues. i, H&E staining for control xenograft tissues; ii, H&E staining for RIPK3-D8 xenograft tissue; iii, immunostaining for control xenograft tissues (×100); iv, immunostaining for RIPK3 overexpression in the xenograft tissues (×100). RIPK3 localized mainly in the cytoplasm of the esophageal cancer epithelial cells.

To further examine the role of RIPK3 in necrosis in ESCC cells, a PCI-RIPK3 expression vector was used to express RIPK3 in KYSE410 cells which was an innate RIPK3-deficient. RIPK3 overexpression was confirmed by western blot analysis after PCI-RIPK3 transfection and the isolation of stable drug-resistant clones (Figure 6A). One of the stable clones, RIPK3-D8, was chosen for analysis to test whether RIP3 overexpression sensitizes KYSE410 cells to cisplatin. As shown in Figure 6B, RIPK3 overexpression significantly increased the cisplatin-induced cell death of KYSE410 cells. To further investigate whether blocking apoptosis enhances cisplatin-induced cell death, Z-LEHD and Z-DEVE, specific inhibitors of caspase-9 and caspase-3, respectively, were used to block apoptosis. Importantly, the addition of Z-LEHD and Z-DEVE did not affect cell death after cisplatin treatment in RIPK3-overexpressing KYSE410 cells, but it significantly reduced cell death in parental cells (Figure 6C). These results suggest that cisplatin induced cell death through programmed necrosis in RIPK3-overexpressing cells after cisplatin treatment. Our observations demonstrated that RIPK3-mediated programmed necrosis plays a partial role during conditions of DNA damage.

### RIPK3 upregulation suppresses *in vivo* xenograft tumor growth

To explore whether overexpression of RIPK3 in the KYSE410 cells affects tumor growth, we assessed the xenograft growth between parental and RIPK3-D8 cells. The size of the RIPK3-overexpressing tumors was significantly reduced compared to the parental KYSE410 tumors (*P*<0.01; Figure 6D). H&E and RIPK3 staining showed a number of necrotic foci in tissue from RIPK3-overexpressing xenograft tumors, and this reduced the tumor size (Figure 6E). These results show that the mitigation in xenograft tumor growth can be attributed to the RIPK3-mediated necrosis in the KYSE410 cells overexpressing RIPK3.

**Figure 7:**
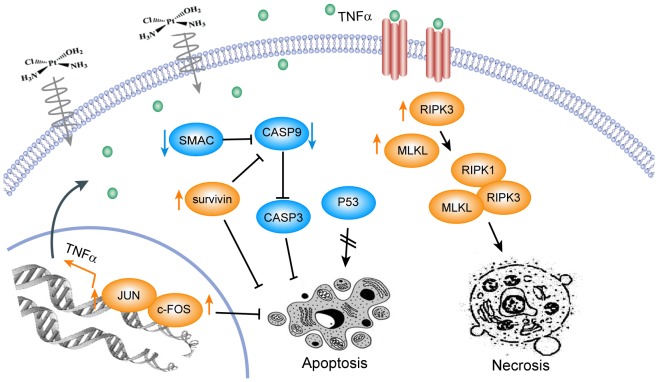
Illustration of the signaling pathway for cisplatin-induced necrosis. Cisplatin-induced necrosis requires both innate suppression of the apoptotic pathway and necrosome assembly, including RIPK1, RIPK3 and MLKL, through autocrine production of TNFα. The blue ellipses represent the downregulated proteins, and the orange ellipses represent the up-regulated proteins.

The above data provide a rationale to better understand the molecular mechanism of cisplatin-induced necrosis. We therefore propose a summary model to explain how cisplatin induces programmed necrosis in ESCC cells, illustrated in Figure 7: (i) promotion of necrosome assembly via autocrine production of TNFα, leading to necrosis, (ii) requirement of RIPK3 for cisplatin-induced necrosis, and (iii) inhibition of endogenous apoptotic pathways. These results showed that RIPK3-mediated programmed necrosis plays a critical role in the suppression of the intrinsic apoptotic pathway and is essential for the efficacy of cisplatin in esophageal cancer chemotherapy.

## Discussion

Although apoptosis has been implicated in cisplatin-mediated cytotoxicity, cisplatin has also been shown to induce non-apoptotic cell death. In recent years, the molecular mechanisms underlying necrosis have been increasingly realized as an important form mechanism of programmed cell death. Necrosis in cancer cells usually occurs when the apoptosis machinery is inhibited or absent [39], [40], [41]. Our previous data indicated that efficient knockdown of pro-apoptotic SMAC protein significantly attenuates the response of esophageal cancer cells to cisplatin [27]. In the present study, we found that cisplatin selectively induces necrosis in KYSE140 cells, which are endogenously deficient in SMAC expression. On basis of the following observations: (i) little nuclear fragmentation and changes in mitochondrial membrane potential were detected in KYSE140 cells in cisplatin-induced cell death; (ii) the release of LDH, ROS production and caspase-independent cell death were observed; and (iii) the visualization of disrupted cytoplasimic membranes using TEM. Further, autocrine production of TNFα, as the pro-death signal, was identified in cisplatin-induced necrotic cell death. This result is consistent with reports showing that autocrine production of TNFα enhanced cellular necrosis in cisplatin-induced acute renal failure [42]. Secretion of endogenous TNFα has been reported to induce downstream signaling, triggering cell death in zVAD-induced necrotic cell death [34]. Thus, our data suggest that cisplatin-induced programmed necrosis in KYSE140 cells is initiated by the autocrine production of TNFα.

Recent advances have elucidated the pathway leading to TNFα-induced programmed necrosis and the core components of the necrosis-inducing necrosome complexes, including RIPK1, RIPK3 and MLKL [25], [36], [43]. Consistent with these results, our data demonstrate that cisplatin-induced programmed necrosis in KYSE140 cells is primarily executed by necrosome formation. The RIPK3/RIPK1/MLKL necrosome complex was increased upon the induction of necrosis, as indicated by immunoprecipitation using anti-RIPK1 and anti-MLKL antibodies in KYSE140 cells. Recently, RIPK3-dependent necrosis has shown to be a common cell-death pathway involved in a variety of pathological conditions [43], [44], [45]. RIPK3 can mediate embryonic lethality in caspase-8-deficient mice through an alternative RIPK3-dependent necrotic cell death pathway [8]. However, it is currently unclear whether RIPK3-mediated necrosis is associated with cisplatin-induced cell death. Our results indicate that RIPK3 is required for cisplatin-induced necrosis in the ESCC cells. This is because (i) mRNA and protein levels of RIPK3 were upregulated after cisplatin treatment; (ii) knockdown of RIPK3 by shRNA prominently attenuated cisplatin-induced necrosis in the SMAC-deficient KYSE140 cells; (iii) the necrotic complex including RIPK1, RIPK3 and MLKL was formed, and the expression levels of its components increased; (iv) overexpression of exogenous RIPK3 still maintained the sensitivity of KYSE410 cells to cisplatin in the presence of caspase inhibitors; (v) RIPK3 overexpression reduced xenograft tumor growth. Our study provides several lines of evidence that RIPK3 plays a key role in cisplatin-induced necrosis and provides new insight into the mechanism of cisplatin-induced necrosis.

It is greatly appreciated that RIPK1 is crucial for necrosome formation and for the execution of necrosis [46], [47]. Recent investigations demonstrated that the formation of the RIPK1-RIPK3 complex is a crucial step in RIPK3 kinase activation, which in turn regulates cellular energy metabolism and the induction of programmed necrosis following stimulation with TNFα or other agonists [17], [24], [25]. Consistent with these findings, our results showed that RIPK1 is necessary for cisplatin-induced necrosis, as both siRNA and pharmacological inhibition of RIPK1 attenuate cisplatin-induced cell death. Thus, using selective knockdown of different individual components of the necrosome, we provide evidence that cisplatin induces necrosis by enhancing the recruiment of the necrosome to excute cell death in ESCC cells. Identification of the mechanism by which RIPK3 regulates cisplatin-induced programmed necrosis may help predict the tumor's response to chemotherapy and radiotherapy.

Although apoptosis is the predominant pathway of cisplatin-induced cell death in cancer cells, necrosis as an alternative pathway is increasingly appreciated for drug cytotoxicity when the apoptotic machinery is suppressed [34], [39], [48]. The current study shows that the apoptotic machinery was attenuated during cisplatin-induced necrosis in KYSE140 cells. This is because (i) the apoptotic response was decreased due to the downregulation of caspase-9, SMAC deficiency, and the lack of caspase-3 activity; and (ii) microarray analysis demonstrated an anti-apoptotic molecular expression pattern involving increased expression of anti-apoptotic genes and reduced expression of pro-apoptotic genes, leading to the suppression of the apoptotic pathway. It is widely known that tumor cells can circumvent apoptosis by increasing expression of anti-apoptotic regulators or by downregulating pro-apoptotic factors [6]. When apoptosis is completely blocked by a caspase inhibitor, such as z-VAD-fmk, RIPK3 is capable of driving necrosis in response to TNFα in some cell types [17], [24], [33]. In our study, microarray data showed that three genes (*FOS, JUN, BIRC5*) associated with the anti-apoptotic response were elevated in the cisplatin-treated esophageal cancer cells. *FOS* and *JUN* are the immediate early genes that are activated in signal transduction systems after different types of stresses, and these genes have been associated with cisplatin resistance through their anti-apoptotic effects [49], [50], [51], [52]. Our data showed that *FOS* was dramatically upregulated in the cisplatin-treated group, as shown by microarray analysis and confirmed by RT-PCR and western blot analyses. We also identified autocrine production of TNFα in supernatants after cisplatin treatment in KYSE140 cells. Previous work has suggested that the TNFα promoter contains AP-1 response elements [53]. Fos, in combination with Jun, forms the AP-1 early response transcription factor in transcriptional regulation. Therefore, it appears that the upregulation of *FOS* and *JUN* may promote the transcription machinery for TNFα expression and autocrine secretion to the medium in cisplatin-induced necrosis in ESCC cells. Our data also showed the up-regulation of proteins such as HSP70, BCL-2 and BIRC5 in the cisplatin-treated esophageal cancer cells, indicating an increase in the anti-apoptotic molecular pattern. Collectively, these data clearly demonstrate that necrosis is an essential complementary mechanism of cisplatin-induced cell death, especially when the apoptotic pathway is blocked.

In summary, our study has several important implications. First, we demonstrated a pathway by which cisplatin triggers programmed necrosis, promoting necrosome assembly via autocrine production of TNFα and leading to necrosis, during inhibition of endogenous apoptotic pathways. Notably, RIPK3 is required for cisplatin-induced programmed necrosis in esophageal cancer cells. Moreover, an anti-apoptotic molecular expression pattern that blocks the apoptotic pathway in response to cisplatin was confirmed by microarray analysis. Our data indicate that RIPK3, necrosomes and autocrine production of TNFα contribute to cisplatin sensitivity by initiating necrosis when the apoptotic pathway is blocked in esophageal cancer cells. A better understanding of the events that activate the necrotic signaling pathway induced by cisplatin is extremely important for improving cancer chemotherapy by exploiting the necrotic machinery. Our results suggest that activation of programmed necrosis is an alternative strategy for killing apoptosis-resistant tumor cells. Sensitivity to necrosis should be considered when defining the susceptibility of individual esophageal cancers to cisplatin.

## Supporting Information

Table S1 **Gene sets associated with canonical biological process.** Canonical biological process of genes up-regulated in KYSE140 after treatment with cisplatin.
(DOC)

Table S2 **Canonical biological process of genes down-regulated in KYSE140 after treatment with cisplatin.**
(DOC)

Table S3 **Description of primers selected for validation by Real-Time RT-PCR.**
(DOC)
