# Supplementary material for: Receptor Interactive Protein Kinase 3 Promotes Cisplatin-Triggered Necrosis in Apoptosis-Resistant Esophageal Squamous Cell Carcinoma Cells
Source: PLoS One. 2014 Jun 24;9(6):e100127. doi: 10.1371/journal.pone.0100127 (PMC4069059; doi:10.1371/journal.pone.0100127)
Supplement: Table S1 — Gene sets associated with canonical biological process. Canonical biological process of genes up-regulated in KYSE140 after treatment with cisplatin. (DOC) [file pone.0100127.s001.doc]

| **Table S1:** Gene sets associated with canonical biological process. Canonical biological process of genes up-regulated in KYSE140 after treatment with cisplatin | | |
| --- | --- | --- |
| **Symbol** | **Genes** |  |
| Positive regulation of transcription | SKIL, ID2, DLX2, EPAS1, JUN, OVOL1, NR4A2, ID2, IL11, GTF2A2, PLAGL1, FOS, NFATC3, CITED2, BIRC5, BCL6, CITED2, CLOCK, MAFB, HEXIM1, MSX1, MAFB, NR4A2 |  |
| Regulation of programmed cell death | PMAIP1, PTGS2, TNFSF13B, JUN, CYCS, NR4A2, HSPA1A, HSPA1B, PLAGL1, MCL1, CDKN1A, NAIP, CITED2, DUSP1, BIRC5, BCL6, PHLDA1, RB1CC1, MSX1, GNRH1, NR4A2 |  |
| Negative regulation of gene expression | SKIL, DLX2, XPO5, CITED2, BCL6, OVOL1, ID2, HEXIM1, NDFIP2, MSX1 |  |
| Programmed cell death | CXCR4, PMAIP1, JUN, AFG3L2, PMAIP1, MCL1, JMJD6, NAIP, CYCS, PHLDA1, NR4A2, JUN, FUS, C3ORF38, RHOB, NR4A2, BIRC5 |  |
| Homeostasis of number of cells | CXCR4, ID2, JUN, AFG3L2, MCL1, JMJD6, CD55, TNFSF13B, EPAS1, BIRC5, BCL6, ALAS2 |  |
| Response to oxygen levels | CITED2, CXCR4, NR4A2, CDKN1A, ALAS2, EPAS1 |  |
| Metabolic process | AOC2, NR4A2, EPAS1 |  |
| Transmembrane receptor protein serine/threonine kinase signaling pathway | FOS, BAMBI, TOB1, MSX1, JUN |  |
| Response to cytokine stimulus | FOS, PTGS2, JUN, MCL1, MMP3 |  |
| Positive regulation of B cell proliferation | BCL6, TNFSF13B, CDKN1A |  |
| Inflammatory response | FOS, CXCR4, CD55, AOC3, NFATC3, LTB4R, MASP2 |  |
