# Supplementary material for: Receptor Interactive Protein Kinase 3 Promotes Cisplatin-Triggered Necrosis in Apoptosis-Resistant Esophageal Squamous Cell Carcinoma Cells
Source: PLoS One. 2014 Jun 24;9(6):e100127. doi: 10.1371/journal.pone.0100127 (PMC4069059; doi:10.1371/journal.pone.0100127)
Supplement: Table S2 — Canonical biological process of genes down-regulated in KYSE140 after treatment with cisplatin. (DOC) [file pone.0100127.s002.doc]

**Table S2:** Canonical biological process of genes down-regulated in KYSE140 after treatment with cisplatin

| **Symbol** | **Genes** |
| --- | --- |
| Positive regulation of biosynthetic process | ZNF462, ZMIZ1, TRERF1, TP63, THRB, THRAP3, TGFB2, TEAD1, TCF7l2, TCF4, TBL1X, STRN3, SOX2, SMARCC1, SMAD3, SATB2, SAMD4A, RXRA, RUNX2, RORA, PTHLH, PPARGC1B, POU2FL, PIAS1, PDGFC, NRIP1, NFIB, NCOA3, NCOA22, NCOA1, MYST4, MYST3, MKL2, MITF, MEIS2, MAML2, LED1, KLF7, KLFL2, ITGA2, IKZF2, IGF1R, HIPK2, HDAC4, GRHL3, GLI3, FOXO1, FOXE1, FAM129A, EGFR, CREB5, BCL11B, ATXN1, ARNT ,ARID1B, ARID1A, ACVR1, ABCA1 |
| Regulation of transcription | ZNF827, ZNF506, ZNF462, ZMYM2, ZMIZ1, ZHX2, ZFP3, ZFHX3, WHSC1L1, TULP4, TRPS1, TRERF1, TP63, TLE4, THRB, THRAP3, TGFBR3, TEAD1, TCF7l2, TCF4, TCF12, TBl1X, TAF4B, TAF4, STRN3, SSBP2, SOX2, SOX1, SND1, SMARCC1, SMAD3, SFMBT2, SATB2, RXRA, RUNX2, RREB1, RORA, RFX7, RBlL, PTPRK, PTHLH, PPARGC1B, POU2F1, PIAS1, NRIP1, NRG1, NRF1, NR3C1, NR2C2, NFIB, NFIA, NFATC1, NCOA3, NCOA2, NCOA1, NACC2, MYST4, MYST3, MLL3, MKL2, MITF, MGA, MEIS2, MED131, MAML2, LMCD1, LIN28B, LEF1, KLF7, KLF12, KDM4C, KDM4B, JMY, JMJD1C, JAZF1, JARID2, IRF2, IKZF2, HMGA2, HIVEP2, HIVEP1, HIPK2, HDAC9, HDAC4, GRLF1, GRHL3, GRHL2, GLI3, FRY, FOXP1, FOXO1, FOXN3, FOXE1, ETV6, ERC1, ELAVL2, EIF2C2, EHMT1, CUX1, CTBP2, CREB5, CHD9, CHD7, CDYL, CBFA2T2, CASZ1, C11ORF30, BRWD1, BCL11B, BCL11A, BBX, BAZ2B, BACH2, ATXN1, ASXL2, ASH1L, ARNTL, ARID5B, ARID4B, ARID4A, ARID1B, ARID1A, APBB2, ACVRL |
| Protein amino acid phosphorylation | AAK1, ACVR1, AKAP9, BCL2, BMPR1A, CAMK2D, CDC42BPA, CDK14, CDK6, CDKL5, CSNK1G1, DAPK1, DYRK1A, EGFR, EPHA4, ERC1, FGD4, FGFR2, FYN, GAB1, GRK5, GSK3B, HIPK2, IGF1R, KSR1, LOC643778, LPAR3, MAP3K4, MAP3K5, MAP4K3, MAP4K4, MARK1, MAST4, NEK6, NTRK2, NUAK1, PIK3CB, PRKCA, PRKCH, PTK2, PXK, ROR1, SGK269, SIK3, SRPK2, STK24, TGFB2, TGFBR3, TRIB2, TRIO, TTBK2 |
| Regulation of small GTPase mediated signal transduction | AGAP1, AKAP13, ARHGEF3, ARHGEF7, ASAP1, C6ORF170, CYTH1, DLC1, DNMBP, FGD4, PLEKHG1, PSD3, RALGAPA1, RAP1GAP2, RAPGEF2, RAPGEF5, RAPGEF6, RASA1, RASA2, RASAL2, RGNEF, SGEF, SIPA1L1, SIPA1L2, TBC1D12, TBC1D8, TBC1D9, TIAM1, TRIO, USP6NL |
| Cell morphogenesis | TGFBR3, TGFB2, SLITRK6, SLIT2, SHROOM3, RXRA, PTK2, PRKCA, NUMB, NRXN3, NRP2, NRP1, NFACTC1, MAP1B, MACF1, LEF1, LAMC1, KLF7, IGF1R, EPHA4, EGFR, EFNA5, DYNC2H1, DST, DLG1, CLASP1, BCL2, BCL11B, APBB2 |
| Cell migration | ACVR1, APBB2, ARHGEF7, ARID5B, FOXE1, FYN, JUB, LAMC1, LRP6, LYST, NCK1, NRP1, NRP2, PPAP2B, PRKCA, PTK2, PTPRK, SATB2, SLIT2, SOX1, TGFB2, TGFBR3, TNS3 |
| Chromatin organization | ARID1A, ARID1B, ARID4A, ARID4B, ASH1L, C11ORF30, CDYL, CHD7, CHD9, EHMT1, HDAC4, HDAC9, HISTLH1A, HMGA2, JMJD1C, KDM4B, KDM4C, KDM6A, MLL3, MYST3, MYST4, NR3C1, RBL1, SATB2, SMARCC1, SOX1, SOX2, WHSC1L1 |
| Embryonic morphogenesis | ACVR1, APAF1, BMPR1A, CHD7, CHST11, DLC1, DYNC2H1, EXT1, FOXE1, GLI3, GNA12, GNAQ, GRLF1, LEF1, LRP6, MACF1, POU2F1, PPAP2B, SATB2, SHROOM3, SLIT2, SMAD3, SOX2, SP8, TCF7l2, TP63, WNT5A |

Up-regulated genes: induced by cisplatin treatment. Down-regulated genes: decreased by cisplatin treatment. Underlined genes are those validated by Real-Time RT-PCR.
