# Supplementary material for: Receptor Interactive Protein Kinase 3 Promotes Cisplatin-Triggered Necrosis in Apoptosis-Resistant Esophageal Squamous Cell Carcinoma Cells
Source: PLoS One. 2014 Jun 24;9(6):e100127. doi: 10.1371/journal.pone.0100127 (PMC4069059; doi:10.1371/journal.pone.0100127)
Supplement: Table S3 — Description of primers selected for validation by Real-Time RT-PCR. (DOC) [file pone.0100127.s003.doc]

**Table S3:** Description of primers selected for validation by Real-Time RT-PCR

|  |  |  |
| --- | --- | --- |
| **Gene symbol** | **Sequence Forward (5' > 3')** | **Sequence Reverse (5' > 3')** |
| JUN | AACAGGTGGCACAGCTTAAAC | CAACTGCTGCGTTAGCATGAG |
| FOS | CACTCCAAGCGGAGACAGAC | AGGTCATCAGGGATCTTGCAG |
| HSPA1A | GCGAGGCGGACAAGAAGAA | GATGGGGTTACACACCTGCT |
| TNF α | CCTCTCTCTAATCAGCCCTCTG | GAGGACCTGGGAGTAGATGAG |
| CYCS | CTTTGGGCGGAAGACAGGTC | TTATTGGCGGCTGTGTAAGAG |
| BIRC5 | AGGACCACCGCATCTCTACAT | AAGTCTGGCTCGTTCTCAGTG |
| NOXA | ACCAAGCCGGATTTGCGATT | ACTTGCACTTGTTCCTCGTGG |
| CASP9 | CTGTCTACGGCACAGATGGAT | GGGACTCGTCTTCAGGGGAA |
| CASP3 | CATGGAAGCGAATCAATGGACT | CTGTACCAGACCGAGATGTCA |
| RIP3 | AATTCGTGCTGCGCCTAGAAG | TCGTGCAGGTAAAACATCCCA |
| APAF1 | GTCACCATACATGGAATGGCA | CTGATCCAACCGTGTGCAAA |
| MLKL | AGGAGGCTAATGGGGAGATAGA | TGGCTTGCTGTTAGAAACCTG |
| GAPDH | CTGGGCTACACTGAGCACC | AAGTGGTCGTTGAGGGCAATG |
